# Multiparametric Ultrasound for Focal Testicular Pathology: A Ten-Year Retrospective Review

**DOI:** 10.3390/cancers16132309

**Published:** 2024-06-24

**Authors:** Dean Y. Huang, Majed Alsadiq, Gibran T. Yusuf, Annamaria Deganello, Maria E. Sellars, Paul S. Sidhu

**Affiliations:** 1 Department of Clinical Radiology, King’s College Hospital, London SE5 9RS, UK; 2 Department of Imaging Sciences, School of Biomedical Engineering and Imaging Sciences, Faculty of Life Sciences and Medicine, King’s College London, London SE1 7EH, UK; 3 Department of Imaging, The Royal London Hospital, London E1 1FR, UK

**Keywords:** multiparametric, ultrasound, testicular cancer, testis-sparing surgery, orchiectomy

## Abstract

**Simple Summary:**

In our retrospective study at a tertiary centre, we reviewed the use of contrast-enhanced ultrasound (CEUS) and strain elastography (SE) as adjuncts to conventional greyscale and colour Doppler US (CDUS) for evaluating focal testicular abnormalities over a decade. This study highlights the potential of advanced ultrasound techniques to provide deeper insights into the characteristics of testicular abnormalities. In particular, we observed that contrast-enhanced ultrasound could detect vascular enhancement in all malignant cases, even those not identified by conventional CDUS, and more conclusively confirm benignity. While SE alone offered no distinctive advantage, incorporating a combination of CEUS and SE into the evaluation of focal testicular abnormalities improved diagnostic performance metrics over conventional CDUS. Our research underscores the enhanced performance achieved by utilising these advanced ultrasound techniques. The comprehensive diagnostic assessment provided by these techniques could facilitate a shift towards more conservative management of testicular lesions, supporting the preference for organ-preserving methods over more radical surgeries.

**Abstract:**

Conventional ultrasonography (US), including greyscale imaging and colour Doppler US (CDUS), is pivotal for diagnosing scrotal pathologies, but it has limited specificity. Historically, solid focal testicular abnormalities often led to radical orchidectomy. This retrospective study evaluated the utilisation of contrast-enhanced ultrasound (CEUS) and strain elastography (SE) in investigating intratesticular focal abnormalities. A total of 124 cases were analysed. This study underscored the superior diagnostic capabilities of CEUS in detecting vascular enhancement in all malignant cases, even those with undetectable vascularity by CDUS. It also highlighted the potential of CEUS in identifying distinctive vascular patterns in benign vascular tumours. Definitive confirmation of benignity could be obtained when the absence of enhancement was demonstrated on CEUS. While SE alone offered no distinctive advantage in differentiating between benign and malignant pathologies, we demonstrated that incorporating a combination of CEUS and SE into the evaluation of focal testicular abnormalities could improve diagnostic performance metrics over conventional CDUS. Our findings underscore the role of advanced ultrasound techniques in enhancing the evaluation of focal testicular abnormalities in clinical practice and could aid a shift towards testis-sparing management strategies.

## 1. Introduction

Conventional ultrasonography (US), including greyscale imaging and colour Doppler US (CDUS), stands as the cornerstone for evaluating scrotal pathologies due to its high resolution, availability, cost-effectiveness, and absence of ionizing radiation [1,2,3,4]. Despite its widespread use, the specificity of greyscale ultrasound in characterising scrotal masses remains limited, often leaving the nature of such lesions ambiguous [5,6,7]. Traditionally, solid testicular lesions, especially those presenting as palpable lumps, have led to radical orchidectomy [8,9]. However, the landscape of scrotal ultrasonography has evolved significantly with advancements in technology and technique, including high frequency, tissue harmonic, and compound imaging. This evolution, alongside a broader spectrum of clinical applications, has increased the detection of small, incidental focal testicular lesions, many of which are benign. Indeed, recent literature suggests a predominance of benignity in these cases, with Leydig cell tumours with low malignant potential (LCT-LMP) constituting a significant fraction among small, impalpable, incidentally discovered testicular nodules [10,11].

This shift in the diagnostic landscape necessitates a reconsideration of radical orchiectomy for focal testicular abnormalities, pivoting towards more organ-sparing approaches when there is a high likelihood of benignity [12]. Yet, despite improved imaging modalities and diagnostic aids, including tumour markers and second-line MRI as recommended by the European Society of Urogenital Radiology (ESUR) [13], significant diagnostic ambiguity persists. This uncertainty complicates the selection of benign lesions for testis-sparing management, underlining a gap in the current diagnostic toolkit.

Contrast-enhanced ultrasound (CEUS) and ultrasound strain elastography (SE) have emerged as valuable adjuncts to traditional ultrasonography, offering insights into vascularisation and tissue elasticity not available through conventional US alone [14,15]. These modalities have shown promise in distinguishing malignant from benign lesions [16,17], guiding management decisions towards more conservative, organ-preserving strategies [18,19,20,21]. Since their adoption in 2008, CEUS and SE have become integral to the multidisciplinary evaluation of testicular abnormalities in our institution [22,23,24,25,26,27,28,29,30,31,32,33,34], marking a significant advance in our approach to scrotal pathology.

The main aim of our retrospective review was to share our decade-long clinical experience with utilising a combination of advanced ultrasound techniques, an approach termed multiparametric ultrasound (MPUS) [18,35], including CEUS and SE, in assessing focal testicular abnormalities. We seek to elucidate how CEUS and SE features correlate with clinical outcomes across a broad spectrum of clinical presentations.

## 2. Materials and Methods

### 2.1. Study Design and Ethical Considerations

This retrospective study assessed the diagnostic accuracy of MPUS in characterising intratesticular focal abnormalities over a ten-year period (2009–2019) at King’s College Hospital, London, United Kingdom. Between 2009 and 2019, 12,981 testicular ultrasound examinations were performed in our department for the following indications: evaluation and location of palpable scrotal masses, detection of primary tumours, follow-up of patients with testicular microlithiasis, follow-up of patients with previous lymphoma, acute scrotum, scrotal trauma, localisation of the undescended testis, detection of varicoceles in infertile men, and evaluation of testicular ischaemia. From an initial dataset of all scrotal ultrasound examinations, 124 consecutive cases of focal testicular abnormalities investigated by MPUS were selected for analysis. Institutional Review Board approval was obtained, with all procedures performed in accordance with ethical standards and patient confidentiality guidelines.

### 2.2. Patient Cohort and Data Acquisition

Eligible cases were identified from the departmental ultrasound database based on the inclusion criteria of having undergone MPUS, comprising greyscale US, CDUS, CEUS, and SE. Comprehensive clinical data, including the patients’ ages, clinical presentations, tumour markers, histopathological reports, and follow-up outcomes, were extracted from electronic patient records. Imaging data were retrieved from the institution’s Picture Archiving and Communication System.

### 2.3. Ultrasound Examination Techniques

The ultrasound examinations were conducted by a team of three radiologists, each with extensive expertise in scrotal ultrasonography and a significant range of experience in MPUS (5–15 years). Prior to the sonographic evaluation, informed verbal consent was obtained from all participants as part of routine clinical practice in our hospital. All scrotal ultrasound studies were conducted utilizing either an Acuson Sequoia (Siemens Mountain View, CA, USA) with a 15L8w transducer or an S2000 system (Siemens Medical Solutions, Mountain View, CA, USA) equipped with either a 14L5 or a 9L4 linear array transducer. Strain elastography examinations were carried out on an HV900 system (Hi-RTE^TM^, Hitachi Medical Corporation, Tokyo, Japan) employing a 14–6 MHz linear transducer and Hitachi real-time tissue elastography.

**Figure 1:**
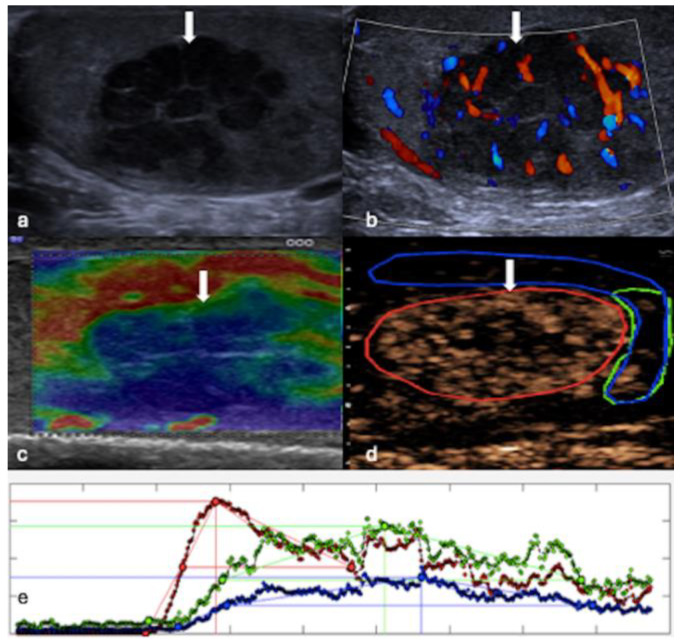
MPUS of a testicular seminoma. (**a**) Greyscale US reveals a large, multiloculated hypoechoic mass (white arrow). (**b**) CDUS demonstrates that the lesion (white arrow) is vascularised. (**c**) On SE, the lesion (white arrow) exhibits uniformly hard tissue stiffness, appearing blue. (**d**) On CEUS the lesion (white arrow) shows enhancement, with late-phase washout evident on the CEUS time–intensity curve (x-axis: time; y-axis: signal intensity) (**e**). Red region of interest (ROI) = lesion; blue and green ROIs = surrounding parenchyma.

**Figure 2:**
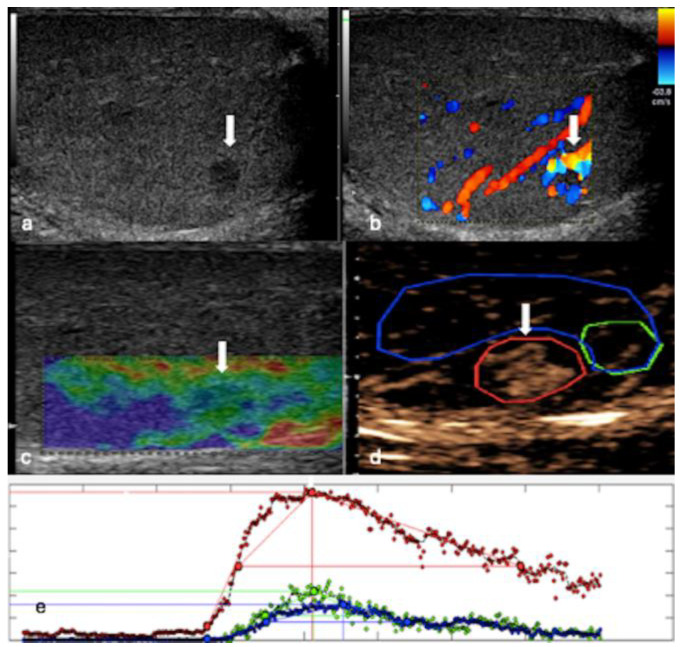
MPUS of a leydig cell tumour. (**a**) Greyscale US shows small, well-defined lesions (white arrow) with homogeneous low reflectivity. (**b**) CDUS indicates that the lesion (white arrow) is highly vascularised. (**c**) SE identifies the lesion (white arrow) as mildly hard, depicted in shades of green and blue. (**d**) CEUS demonstrates a hyper-enhancing lesion (white arrow), with prolonged hyper-enhancement relative to the surrounding parenchyma in the late phase on the CEUS time–intensity curve (x-axis: time; y-axis: intensity) (**e**). Red region of interest (ROI) = lesion; blue and green ROIs = surrounding parenchyma.

**Figure 3:**
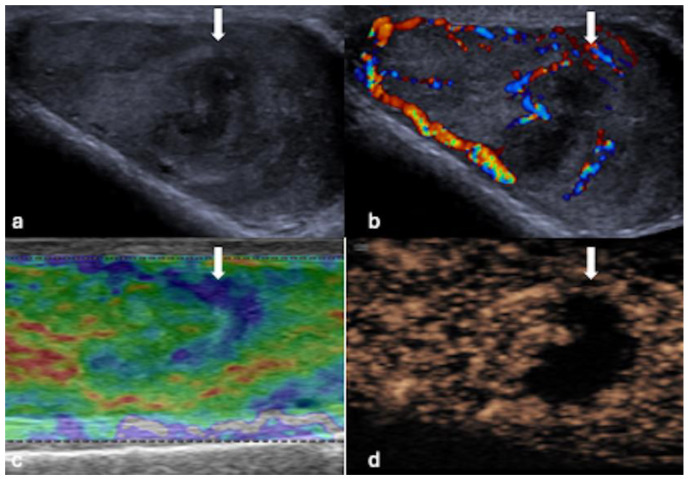
MPUS of a segmental infarction. (**a**) Greyscale US shows a lesion (white arrow) with a low echogenic centre and surrounding high echogenicity. (**b**) CDUS indicates that no colour Doppler signal is present within the lesion (white arrow). (**c**) On SE, this lesion (white arrow) demonstrates a predominantly green signal consistent with a “soft” lesion. (**d**) CEUS conclusively demonstrates the absence of enhancement within the central aspects of the lesion (white arrow).

**Figure 4:**
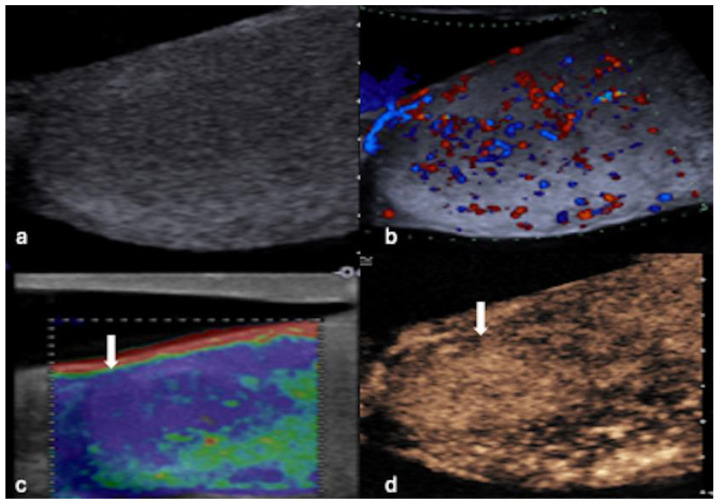
MPUS of a testicular lymphoma. (**a**) Greyscale ultrasound shows diffuse enlargement of the testis with ill-defined, extensive decreased echogenicity in the majority of the testis. (**b**) CDUS indicates that hypervascularity is present within the testis. (**c**) SE demonstrates a hard lesion (white arrow), which is not clearly depicted on greyscale US. (**d**) CEUS demonstrates hyper-enhancement of the lesion (white arrow).

Scrotal ultrasound was performed with the patient in the supine position, holding the penis lifted onto the abdomen and covered. Standardised greyscale US pre-sets were used with abnormalities imaged in both axial and longitudinal planes in accordance with established protocol [2]. The operators varied the pulse repetition frequency, focal zone, gain, and wall filter as necessary to obtain optimal sonograms in each case. Colour Doppler ultrasound was performed with the highest signal gain setting possible without the appearance of background noise and low pulse-repetition frequencies (0.2–0.4) to maximise sensitivity to slow flow velocities. CEUS and SE assessments were undertaken following the identification of a focal intratesticular abnormality via initial greyscale and CDUS evaluations as part of our clinical practice. CEUS examinations employed bolus injection of ultrasound contrast agents for contrast administration. Harmonic imaging with a low-mechanical index technique (Cadence contrast pulse sequencing (CPS^TM^); Siemens Medical Solutions, Mountain View, CA, USA) was utilised, setting the mechanical index at or below 0.10, typically implemented at 10–20 frames per second during the enhancement phase. A bolus injection of 4.8 mL of SonoVue^TM^ (Bracco SpA, Milan, Italy), a sulphur hexafluoride microbubble contrast agent, was administered, followed by a 10 mL normal saline flush via a 20-gauge cannula inserted in the antecubital vein. During CEUS examinations, one examiner maintained the transducer over the area of interest while a second radiologist administered the ultrasound contrast agent. Continuous observation was performed from the time of arrival of the microbubbles for at least 90 s after injection of ultrasound contrast agent in the majority of cases. All utilised ultrasound systems featured dual-screen display capabilities, enabling the simultaneous presentation of the underlying modified greyscale image alongside the CEUS image, allowing the operator to retain the interrogated abnormality within the field of view throughout the entire examination. Ultrasound SE examinations were conducted in real time using a freehand technique. Each abnormality was assessed by applying gentle pressure, which was adjusted according to the on-screen quality indicator scale for compression strain. The stiffness of the abnormality was compared to the surrounding tissue and visually represented through colour coding, with the stiffest areas depicted in blue, the softest tissues in red, and areas of intermediate elasticity in green to yellow on the display. All static images and cine loops were preserved within our picture archiving and communication system (PACS, Centricity, GE Healthcare, Germany) or in our institution’s upgraded picture archiving and communication system (SECTRA, Linköping, Sweden) (Figure 1, Figure 2, Figure 3 and Figure 4).

### 2.4. Clinical Decision Making and Intervention

Management decisions for identified focal intratesticular abnormalities were formulated by a multidisciplinary team comprising urologists, oncologists, radiologists, and histopathologists. Criteria for radical orchiectomy included definitive malignant ultrasound features, elevated tumour markers, and evidence of metastasis. Conversely, lesions considered likely benign were managed with either surveillance or testis-sparing surgery (TSS). Criteria for TSS selection encompassed abnormality size under 2 cm, a safe distance between the mass and rete testis, negative tumour markers in patients, and the absence of metastatic disease ascertained by computed tomography staging evaluation.

The standard surgical technique for managing intratesticular abnormalities is the inguinal approach. In the context of TSS, the procedure involves exteriorisation of the testis followed by incision of the tunica vaginalis to facilitate direct examination. Localisation of the tumour is achieved either through manual palpation or via intraoperative ultrasound guidance. Additionally, intraoperative frozen section examinations are employed at the discretion of the surgical team to aid in immediate histopathological evaluation. 

### 2.5. Data Analysis

Two experienced reviewers, each with over five years of expertise in scrotal ultrasonography and blinded to the other’s evaluations, independently recorded the ultrasound features of each lesion. To ensure the objectivity and integrity of our data analysis, both reviewers were completely blinded to all clinical information, including the presence or absence of raised tumour markers and distant metastasis. The reviewers’ assessments were based solely on the imaging data presented to them, devoid of any preconceived notions about the patients’ clinical status. Discrepancies were resolved through joint discussions between the reviewers to reach a consensus. The assessment focused on documenting essential sonographic characteristics, such as size, echogenicity, vascular patterns, contrast enhancement properties, and strain elastography findings. Strain elastography results were analysed using a colour-coded scheme to delineate varying degrees of tissue stiffness in accordance with established criteria for testicular strain elastography [26,36]. 

**Figure 5:**
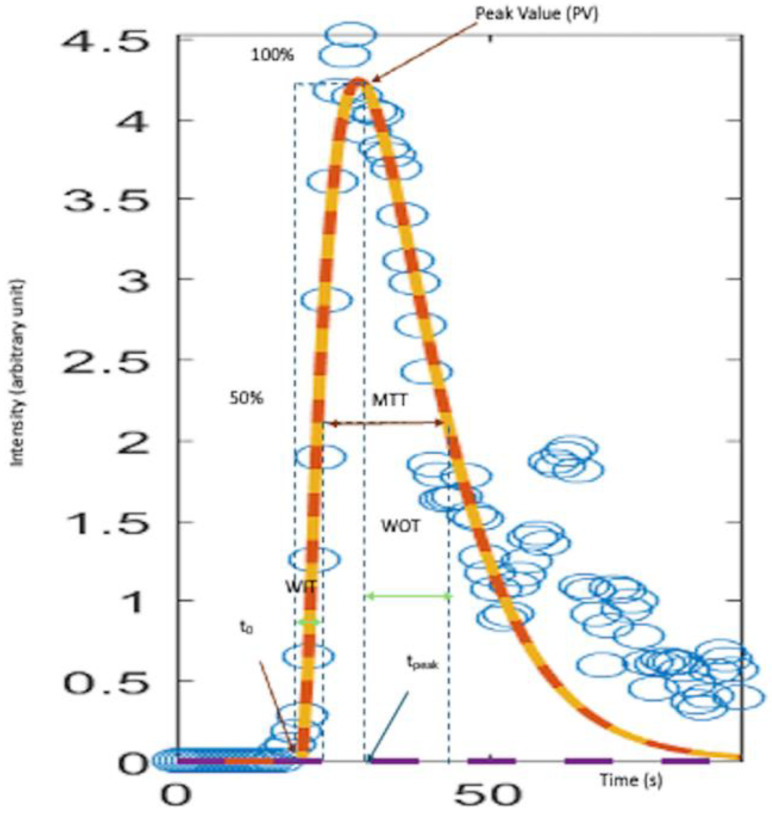
Quantitative CEUS analysis of an intratesticular abnormality. Perfusion parameters derived from the time–intensity curve (blue circles: intensity data entries) with curve-fitting (orange curve) include the following: tmax (time to peak, TTP): time needed from contrast injection to maximum intensity (s); peak value (PV): the maximum intensity on the TIC curve (arbitrary units, arb); wash-in time (WIT) (raise time): time from 5% intensity to 50% intensity (s); wash-out time (WOT): time from the peak of the TIC curve to the 50% PV value (s); inflow rate (5 s): calculated as the rate of rise in the first 5 s from t0 (arb/s); inflow rate: calculated as the rate of rise over tpeak-t0 (arb/s); outflow rate (5 s): calculated as the rate of outflow in the first 5 s from PV (arb/s); outflow rate: calculated as peak enhancement divided by WOT for the descending slope to reach a contrast signal intensity of zero or the end of the curve (arb/s); MTT (mean transit time, full width at half maximum, FWHM): The time between the half-amplitude values on each side of the maximum (s).

Quantitative CEUS of time–intensity curves (TICs) was performed to evaluate perfusion parameters, facilitating a comparative study between histologically verified benign and malignant lesions, including specific analysis within the two largest homogeneous groups of malignant seminomas and benign Leydig cell tumours with low malignant potential. Exclusions from the TIC analysis were made in instances of compromised image loop storage, alterations in imaging planes, or adjustments in receiver gain settings. Pixel intensity data, expressed in grey levels, were converted to echo-power units (arbitrary units, au) using MATLAB (version R2015a), facilitating a more precise analysis of the echo intensity within the region of interest (ROI). This conversion was followed by the application of a gamma variate curve fitting to the raw data, from which various perfusion parameters were calculated based on the fitted model (Figure 5).

### 2.6. Reference Standards and Diagnostic Criteria

Final diagnoses were confirmed either through histopathological examination of specimens obtained via surgical intervention or through a minimum follow-up period of twelve months for non-surgical cases. Lesions were identified as malignant based on the presence of malignant histological features in the analysed specimens, including instances of burnt-out tumours. Conversely, lesions were classified as benign following histopathological analysis of excised material or orchiectomy specimens that confirmed benign histological characteristics, including Leydig cell tumours with low malignant potential, as delineated by specific histological criteria [37,38,39]. Furthermore, lesions demonstrating no change, stability, or reduction in size during follow-up were also classified as benign.

### 2.7. Statistical Analyses

Quantitative data were presented as either mean ± standard deviation for normally distributed variables or median and interquartile range for variables not following a normal distribution. For participants presenting with multiple testicular lesions, the largest abnormality was selected for inclusion in the statistical comparison of ultrasound features between benign and malignant categories. The Mann–Whitney U test was applied to evaluate significant differences in continuous variables across the two groups. Interobserver reliability was quantified using Cohen’s kappa coefficient for the two independent reviewers. Variations in categorical data between benign and malignant lesions were assessed using Pearson’s Chi-squared test and Fisher’s exact test as appropriate. The McNemar test was used to compare sensitivity, specificity, and accuracy, and bootstrapping was used to quantify appropriate confidence intervals and obtain the significance of the difference for positive predictive values (PPVs) and negative predictive values (NPVs) between groups. The DeLong test was used to compare the area under the curve (AUC) for the receiver operating characteristic (ROC) curves between ultrasound techniques. Logistic regression analysis was conducted to examine the influence of specific sonographic features on the probability of a lesion being malignant, utilising Nagelkerke R^2^ and the Hosmer and Lemeshow test as measures of goodness-of-fit. The Wilcoxon signed-rank test was used for comparing perfusion parameters between each lesion and its adjacent normal testicular tissue. All statistical analyses were performed using SPSS software (version 29, IBM), with a significance threshold set at *p* < 0.05.

## 3. Results

A total of 124 MPUS examinations conducted to evaluate intratesticular focal abnormalities were included. This cohort comprised 78 benign and 46 malignant diagnoses. The demographic analysis (Table 1) demonstrated no significant age difference between patients with benign and those with malignant testicular abnormalities (*p* = 0.27). A significant difference (*p* = 0.005) was observed in the palpability of the focal abnormalities, with a greater prevalence in malignant cases compared to benign cases. Elevated tumour markers were noted only in 9 out of 46 patients with malignant lesions and none in patients with a benign diagnosis. 

Histopathological analysis was available for 76 cases (Table 2), identifying 46 as malignant and 30 as benign. Among these, 16 patients underwent testis-sparing surgery, with all abnormalities in this subset confirmed as benign. For the remaining 48 cases not undergoing surgery, benignity was determined based on observed stability or regression during the follow-up period.

### MPUS Characteristics

The sonographic features observed are summarised in Table 3. High interrater reliability was demonstrated, with a Cohen’s kappa coefficient of 0.96 for greyscale ultrasound, 0.94 for CDUS, 0.96 for CEUS, and 0.93 for strain elastography. The average dimension of the focal abnormalities was 15.26 ± 13.20 mm. A significant difference (*p* < 0.001) in size was observed, with benign abnormalities measuring 10.02 ± 6.80 mm and malignant abnormalities 21.12 ± 12.19 mm. Among the 124 evaluated abnormalities, 93 (75.0%) presented as predominantly hypoechoic on greyscale ultrasound, with three abnormalities being isoechoic and two hyperechoic. Additionally, 26 abnormalities (21.0%) demonstrated mixed echogenicity. No significant differences were detected between benign and malignant categories concerning the hypoechoic appearance, the presence of macro-calcification, or irregular margins. Similarly, the incidence of testicular microlithiasis (TML) did not significantly differ between the groups. 

Vascularisation, as detected by CDUS, was observed in 89.10% (41/46) of malignant abnormalities and 52.56% (41/78) of benign abnormalities, yielding a statistically significant difference (*p* < 0.001). Nonetheless, CDUS did not detect vascularisation in 10.87% (5/46) of malignant abnormalities, including three seminomas, one mixed germ cell tumour, and one burnt-out tumour. During contrast-enhanced ultrasound (CEUS) examination, enhancement was observed in all malignant abnormalities (46/46, 100%) but also in 37.18% (49 out of 78) of benign abnormalities. Notably, CEUS detected enhancement in all 5 malignant abnormalities, which were initially characterised as ‘avascular’ by CDUS, while all 29 abnormalities lacking enhancement on CEUS were benign. Within the subset of 95 abnormalities demonstrating enhancement on CEUS, there were no statistically significant differences between benign and malignant groups regarding homogeneous enhancement or early (within 40 s of contrast injection) hyperenhancement. However, a significant distinction was observed in late (40–90 s post-contrast injection) hyperenhancement on CEUS. Additionally, in comparing the two largest histologically verified homogeneous groups of vascular tumours—malignant seminomas and LCT-LMP—significant differences (*p* = 0.04) in late hyperenhancement between the two groups were observed, with seminomas (5/29, 17.24%) and LCT-LMP (8/17, 47.06%) demonstrating late hyperenhancement. Strain elastography revealed no significant differences in increased tissue stiffness between benign and malignant abnormalities. Likewise, comparisons of stiffness between seminomas and LCT-LMP showed no significant differences, with increased stiffness observed in 13 out of 17 LCT cases and 27 out of 29 seminomas during strain elastography analysis (*p* = 0.174). A subset of 53 lesions smaller than 10 mm, comprising 7 malignant and 46 benign lesions, was also analysed (Table 3). No statistically significant differences were noted between the benign and malignant groups in terms of margin, presence of microlithiasis, CDUS vascularity, CEUS enhancement, the presence of prolonged hyperenhancement on CEUS, or stiffness on SE. However, it is worth noting that two out of the seven malignant lesions displayed no vascularity on CDUS. Both of these malignant lesions showed enhancement on CEUS, and all lesions < 10 mm that demonstrated no enhancement were benign. 

The comparative analysis of imaging modalities, including conventional CDUS, CEUS, SE, and their combined application (CEUS+SE), is summarised in Table 4. CEUS achieved the highest sensitivity but had a low specificity of 37.18% (95% CI: 26.50 to 48.87). In terms of specificity, the combination of CEUS and SE showed the best performance at 60.26% (95% CI: 48.54 to 71.17), which was significantly higher than conventional CDUS (*p* = 0.04). Although it did not reach statistical significance when compared to CDUS (*p* = 0.12), the accuracy rate of the combined CEUS and SE approach (70.16%, 95% CI: 61.29 to 78.04%) suggests an improvement compared to CDUS (62.90%, 95% CI: 53.77 to 71.40%), CEUS (60.48%, 95% CI: 51.31 to 69.14%), or SE (54.84%, 95% CI: 45.65 to 63.79%). Other diagnostic performance metrics did not show significant differences among these diagnostic tests.

This table summarises the performance metrics (values and respective 95% confidence interval) of CDUS, CEUS, SE, and CEUS + SE. For CDUS, the presence of hypervascularity indicates malignancy. For CEUS, the presence of enhancement indicates malignancy. For SE, hardness indicates malignancy. For CEUS + SE, the presence of both enhancement and hardness indicates malignancy. TP = true positive, FP = false positive, TN = true negative, and FN = false negative. LR = likelihood ratio. PPV = positive predictive value. NPV = negative predictive value. AUC = area under the ROC curve. 

A multivariable logistic regression analysis (Table 5) was conducted to evaluate the contribution of various sonographic features identified as independent predictors of malignancy in abnormalities enhanced on CEUS. Investigated factors encompassed lesion size larger than 10 mm, homogeneous enhancement, early hyperenhancement, absence of late hyperenhancement, and increased tissue stiffness as determined by SE. The model demonstrated significant predictive capability (Nagelkerke R^2^ = 0.49), fitting the data well (χ^2^(6) = 5.31, *p* = 0.50), correctly classifying 75.50% of benign cases and 84.80% of malignant cases, with an overall accuracy of 80.00%. Within this model, two features had a statistically significant effect on the outcome: lesion size larger than 10 mm and absence of late hyperenhancement. The findings of a lesion size larger than 10 mm had a highly significant effect on the outcome (*p* < 0.001), with an odds ratio (OR) of 9.72 (95% CI: 2.97 to 31.86), indicating that for an enhancing lesion, having a size larger than 10 mm increases the odds of malignancy by nearly ten times. The absence of late hyperenhancement on CEUS was also significant (*p* = 0.01) with an OR of 5.81 (95% CI: 1.43 to 23.65), suggesting that for an enhancing abnormality, the absence of late hyperenhancement increases the odds of malignancy by approximately six times.

In the comparative quantitative analysis of perfusion parameters derived from time–intensity curves during CEUS evaluations, normalisation of data relative to the surrounding parenchyma (lesion-to-parenchyma ratios) revealed no statistically significant differences in all parameters between benign and malignant groups (Table 6). Nonetheless, a notable distinction was identified in the perfusion parameters between histologically confirmed seminomas and LCT-LMP: seminomas exhibited a significantly shorter washout time compared to the adjacent parenchyma (35.30 ± 6.61 s vs. 44.88 ± 15.23 s; *p* = 0.03), in contrast to LCT-LMP, which did not show a statistically significant difference in washout time to the surrounding parenchyma (25.13 ± 12.20 s vs. 34.12 ± 16.90 s, *p* = 0.25).

## 4. Discussion

In this study from a tertiary centre, we describe our decade-long experience with multiparametric ultrasound, including CEUS and SE, in evaluating focal testicular abnormalities. To our knowledge, this series represents the largest cohort published to date [18,40,41,42]. We evaluated the contribution these techniques bring to clinical practice. All malignant lesions demonstrated enhancement on CEUS, including 10.8% of malignant tumours that were deemed ‘avascular’ on CDUS in our cohort. Conversely, lesions without CEUS enhancement were uniformly benign. Our cohort also revealed that late hyperenhancement on CEUS in benign enhancing tumours, such as in Leydig cell tumours with low malignant potential, offers a potential feature for distinguishing these from malignant tumours, such as seminomas. This study also showed that by integrating CEUS and the combination of CEUS and SE, the diagnostic performance of ultrasound imaging in differentiating benign and malignant focal testicular abnormalities was improved. Specifically, CEUS showed a higher sensitivity when compared to CDUS, and CEUS+SE showed higher specificity when compared to CDUS. Notably, during our decade-long clinical experience, all patients who opted for testis-sparing surgery instead of radical orchiectomy following MPUS assessments were confirmed to have benign conditions.

In our study, a statistically significant difference was observed in the association of lesion size and final diagnosis between the benign and the malignant groups in our cohort of patients. In a study by Eifler et al. [43] based on 49 lesions in 145 men referred for azoospermia who underwent ultrasonographic analysis, the investigators proposed an algorithm based on tumour markers and the size and vascularity of the lesions. They suggested that a lesion < 5 mm, characterised by an absence of vascularity and negative tumour markers, could be followed by serial US monitoring. A further study by Scandura et al. [44] reported the majority of testicular lesions < 10 mm identified by radiology were benign. In our study, it was shown that for an enhancing lesion, having a size larger than 10 mm increases the odds of malignancy by nearly ten times. However, we found that greyscale ultrasound did not demonstrate statistically significant differences in features such as margin, hypoechoic nature, or the presence of microlithiasis between benign and malignant testicular lesions. The lack of significant differences in these observed greyscale sonographic features suggests that these parameters alone are insufficient for reliable differentiation, underscoring the limitation of greyscale US.

The descriptive statistical analysis of various ultrasound modalities, including CDUS, CEUS, and SE, demonstrated high sensitivity across these techniques, aligning with findings reported in existing literature [16,18,45,46]. However, all examined modalities exhibited low specificity in our study, with SE identified as the least specific technique. Existing research on the diagnostic accuracy of SE for evaluating testicular lesions presents divergent outcomes [46,47,48,49]. For instance, Grasso et al. [50] compared B-mode plus colour Doppler ultrasound to real-time elastography (RTE) in 41 patients and noted the inability to differentiate malignant from benign lesions based solely on elastography. Goddi et al. [48] reported an SE sensitivity of 87.5% with a specificity of 98.2% in a large series of 144 testicular lesions but comprising a clear majority of benign lesions (112 of 144, 77%). Aigner et al. [47] reported similar sensitivity (100%) and specificity (81%) in 62 patients. Conversely, Marsaud et al. [51] subsequently reported a sensitivity for strain elastography to be 96% in 34 patients (26 malignant), but specificity proved to be as low as 37.5%. Schrodeer et al. [46] reported a specificity of 25%, with only one-quarter of non-neoplastic lesions being correctly identified by SE in their study. Our findings support the notion that SE, when used alone, is not definitive in distinguishing between malignant and benign abnormalities. This observation aligns with current guidelines regarding the use of elastography in evaluating testicular focal abnormalities [15]. However, our data indicate that SE could still provide added value in diagnosing focal testicular abnormalities. In our cohort, combining SE with CEUS significantly improves specificity compared to using conventional CDUS alone.

In our study, CEUS exhibited high sensitivity for intratesticular malignant tumours (100%). Notably, CEUS identified enhancement in all malignant lesions across our cohort, including instances where CDUS could not ascertain tissue vascularisation, such as malignant lesions < 10 mm. This reinforces the notion that CEUS offers superior capabilities in depicting vascular flow [14]. Although CDUS is widely used in the evaluation of most intratesticular tumours, the technique is not without limitations. Ma et al. [52] showed that a substantial proportion (36.5%) of hypoechoic testicular lesions that were avascular on CDUS were malignant in their cohort. Our findings, therefore, underscore the distinctive clinical advantages of CEUS over CDUS. The adjunctive application of CEUS facilitates the timely identification of testicular malignancies, which may otherwise not exhibit a vascular signal on CDUS, thereby mitigating the risk of diagnostic delays. The importance of not missing any tumour is crucial not only for immediate treatment outcomes but also for long-term prognosis and survival, particularly in the context of malignancies where early detection can significantly influence the treatment approach and outcome [53]. 

Our study determined that lesions exhibiting no enhancement on CEUS were invariably benign, lending credence to the interpretation that the absence of vascularity on CEUS is a robust indicator of benignity, as supported by the low incidence of false negatives in related research with CEUS [16,20]. Our finding substantiates the view that lack of vascularity on CEUS can be interpreted as a strong indicator of benignity. The confirmation of the absence of vascularisation with CEUS excludes (‘rule out’) a malignant diagnosis more reliably than CDUS. Examples of such cases include epidermoid cysts and hard infarctions, which can be conclusively considered to be benign when no internal enhancement is demonstrated on CEUS. In practical terms, this finding indicates that adjunct CEUS allows increased diagnostic confidence when a focal intratesticular lesion is encountered in routine urological practice, allowing accurate triaging of patients for conservative management, such as watchful waiting or testis-sparing surgery, versus the alternative of an unnecessary orchiectomy, in a clinically appropriate setting. 

A significant portion of our cohort featured vascular benign abnormalities, such as LCT-LMP. This prevalence of vascular benign lesions contributed to misclassifications by both CDUS and CEUS when the detection of increased perfusion in lesions is solely relied on as a binary marker for malignancy. However, our findings indicate that for an enhancing abnormality, the absence of late hyperenhancement raises the likelihood of malignancy by approximately six times, highlighting the unique benefits of CEUS. While the investigation of tissue vascularisation is not the prerogative of CEUS alone, in our investigation, CEUS exhibited the capability to identify a distinct vascular pattern of prolonged enhancement, potentially aiding in the differential diagnosis of benign vascular testicular lesions, such as LCT-LMP, a finding corroborated by other groups [16,54]. These observations in our study hold significant clinical implications, as small incidental Leydig cell tumours are more likely to have a benign course [55,56], and distinct vascular patterns for LCT-LMP on CEUS may inform decisions to forego orchiectomy in favour of more conservative organ-sparing approaches for vascular focal lesions presumed to be LCT-LMP [57,58], contingent upon clinical judgment (e.g., negative tumour markers, normal staging computed tomography, and patient suitability). 

All patients who underwent testis-sparing surgery in our cohort had benign diagnoses. In clinical settings, imaging findings, along with clinical risk and biochemical assessment, allow increased confidence for the most appropriate clinical management pathway to be instituted by the multi-disciplinary team caring for patients. If a testicular lesion can be shown to be of a high probability to be benign pre-operatively, the organ-sparing approach to small testicular lesion represents a valid treatment, as testis-sparing surgery can provide accurate histological diagnosis and optimal oncological efficacy yet preclude the risk of removal of a testicle bearing a benign lesion. Muller et al. [59] reported their experience in testis-sparing surgery for incidentally detected testicular lesions < 5 mm. All patients who opted for testis-sparing surgery instead of radical orchiectomy following MPUS assessments were confirmed to have benign conditions during our decade-long clinical experience. Our experience showed the utility of advanced ultrasound techniques in preoperative characterisation could aid in facilitating the formulation of optimal clinical management strategies. 

This study is subject to some limitations. Primarily, it was structured as a retrospective analysis, which may impact the prospective applicability of the findings. Our study encompasses a wide range of cases encountered in routine clinical practice at our tertiary care centre over a ten-year period. Our dataset includes clinically impalpable lesions incidentally discovered in adult men presenting with symptoms such as scrotal pain or subfertility. Including both palpable and impalpable lesions allows us to assess the effectiveness of ultrasound in a realistic clinical setting and reflect the heterogeneity observed in routine practice. However, this broad inclusion complicates the extraction of precise guidance for populations with specific clinical presentations. The series size was modest, covering a wide spectrum of testicular pathologies but with limited cases per specific condition, such as malignant lesions < 10 mm. This suggests a need for future studies on a larger scale to validate these results with greater statistical power. Additionally, this study focused on a select group of patients with focal testicular abnormalities identified through greyscale ultrasound, risking selection bias. The operator-dependent nature of ultrasound also presents a challenge in ensuring consistent and reproducible results across different examiners. Lastly, non-surgical cases managed as benign were not confirmed histologically, leaving room for diagnostic uncertainty. 

## 5. Conclusions

Our decade-long experience indicates that integrating advanced ultrasound technologies, such as contrast-enhanced ultrasound and strain elastography, could refine the diagnosis of focal intratesticular abnormalities. Future research should continue to explore and validate the clinical benefits of these technologies, aiming to establish clear protocols that optimise their use in everyday medical practice.

## Figures and Tables

**Table 1:** Summary of clinical features.

	Malignant	Benign	*p*-Value Benign vs. Malignant
Number of patients	46	78	
Age (mean ± standard deviation)	37.13 ± 10.29	39.55 ± 23.51	*p* = 0.27
Site (number (%))			
Right	28 (60.87%)	33 (42.31%)	*p* = 0.05
Left	17 (36.70%)	39 (50.00%)	*p* = 0.16
Bilateral	1 (2.17%)	6 (7.69%)	*p* = 0.20
Clinical Presentation (number (%))			
Palpable lump	29 (63.04%)	29 (37.18%)	*p* = 0.005
Pain	7 (15.22%)	24 (30.77%)	*p* = 0.05
Trauma	0	6 (7.69%)	*p* = 0.05
Inflammatory	2 (4.34%)	3 (3.85%)	*p* = 0.89
Infertility	0	3 (3.85%)	*p* = 0.18
Post-surgery	0	2 (2.56%)	*p* = 0.27
Asymptomatic	8 (17.39%)	11 (14.10%)	*p* = 0.62
Positive Tumour Markers (number (%))	9 (19.57%)	0	*p* < 0.001
Clinical Management (number, (%))	Orchiectomy: 46 (100%)	Orchiectomy: 14 (17.94%) Testis-sparing surgery: 16 (20.51%) Follow-up: 48 (61.54%)	

**Table 2:** Histopathological analysis of 76 surgical cases.

Variables	Orchiectomy	Testis-Sparing Surgery
Number of patients	60	16
Histological diagnosis: Malignant		
Seminoma	29	0
Mixed germ cell tumours	12	0
Lymphoma	2	0
Burnt-out tumour	1	0
Metastasis (prostate primary)	1	0
Sarcoma	1	0
Total	46	0
Histological Diagnosis: Benign		
Leydig cell tumour with low malignant potential	7	9
Sertoli cell tumour	0	1
Global testicular infarct	6	0
Abscess	2	0
Adenomatoid tumour (intratesticular)	1	1
Epidermoid cyst	1	1
Fibrotic change	1	1
Calcified haematoma	0	1
Lipomatous hamartoma	1	0
Inflammatory reaction	1	0
Lobular capillary haemangioma	1	0
Sarcoidosis	0	1
TB	1	0
Mature teratoma	1	1
Total	14	16

**Table 3:** Summary of comparative MPUS features between the benign and malignant groups.

MP-US Features	Benign (All Lesions, *n* = 78)	Malignant (All Lesions, *n* = 46)	*p*-Value (All Lesions, Benign vs. Malignant)	Benign (<10 mm, *n* = 46)	Malignant (<10 mm, *n* = 7)	*p*-Value (<10 mm, Benign vs. Malignant)
Maximal dimension (mm)	10.02 ± 6.80	21.12 ± 12.19	*p* < 0.001	5.25 +/- 2.25	5.59 +/- 2.55	*p* = 0.72
Echogenicity			*p* = 0.83			*p* = 0.31
Not hypoechoic	20	11		6	0	
Hypoechoic	58	35		40	7	
Margin			*p* = 0.65			*p* = 0.92
Well-circumscribed	49	27		32	5	
Poorly circumscribed	29	19		14	2	
Testicular Microlithiasis			*p* = 0.89			*p* = 0.79
Not present	71	37		41	6	
Present	7	9		5	1	
CDUS vascularity			*p* < 0.001			*p* = 0.67
Not present	37	5		17	2	
Present	41	41		29	5	
CEUS enhancement			*p* < 0.001			*p* = 0.17
Not present	29	0		10	0	
Present	49	46		36	7	
CEUS homogeneous enhancement			*p* = 0.08			*p* = 0.52
Heterogenous enhancement	7	14		2	0	
Homogeneous enhancement	42	32		34	7	
CEUS early hyperenhancement			*p* = 0.72			*p* = 0.83
Not present	22	19		14	4	
Present	27	27		22	3	
CEUS late hyperenhancement			*p* = 0.002			*p* = 0.44
Not present	29	40		20	5	
Present	20	6		16	2	
Strain elastography			*p* = 0.07			*p* = 0.52
Soft	28	6		19	2	
Hard	50	40		27	5	

**Table 4:** Performance metrics of CDUS, CEUS, and SE.

	CDUS	CEUS	*p*-Value CDUS vs. CEUS	SE	*p*-Value CDUS vs. SE	CEUS + SE	*p*-Value CDUS vs. CEUS + SE
TP	41	46		40		40	
FP	41	49		50		31	
TN	37	29		28		47	
FN	5	0		6		6	
Sensitivity (%)	89.13 (76.43–96.38)	100.00 (92.29–100.00)	*p* = 0.004	86.96 (73.74–95.06)	*p* = 0.28	86.96 (73.74–95.06)	*p* = 0.05
Specificity (%)	47.44 (36.01–59.07)	37.18 (26.50–48.87)	*p* = 0.06	35.90 (25.34–47.56)	*p* = 0.18	60.26 (48.54–71.17)	*p* = 0.04
PPV (%)	50.00 (39.20–61.10)	48.42 (38.20–58.60)	*p* = 0.33	44.44 (34.70–54.70)	*p* = 0.10	56.34 (45.20–67.50)	*p* = 0.09
NPV (%)	88.10 (78.80–96.30)	100 (100.00–100.00)	*p* = 0.12	82.35 (69.00–94.10)	*p* = 0.27	88.68 (78.80–96.30)	*p* = 0.49
Accuracy (%)	62.90 (53.77–71.40)	60.48 (51.31–69.14)	*p* = 0.79	54.84 (45.65–63.79)	*p* = 0.25	70.16 (61.29–78.04)	*p* = 0.12
AUC	0.68 (0.61-0.76)	0.69 (0.63–0.74)	*p* = 0.93	0.61 (0.54–0.69)	*p* = 0.15	0.74 (0.66–0.81)	*p* = 0.19

**Table 5:** Multivariable logistic regression analysis to evaluate the contribution of various features identified via CEUS and SE as independent predictors of malignancy.

Sonographic Features	ß Coefficient	Standard Errors	*p*-Value	OR	95% Confidence Intervals for OR	
					Lower	Upper
Lesion size > 10 mm	2.27	0.61	*p* < 0.001	9.72	2.97	31.86
Homogeneous enhancement	0.12	0.69	*p =* 0.86	1.13	0.29	4.33
Early hyperenhancement	0.84	0.62	*p =* 0.18	2.32	0.68	7.90
Absence of late hyperenhancement on CEUS	1.76	0.72	*p =* 0.01	5.81	1.43	23.65
Increased tissue stiffness on SE	1.03	0.69	*p =* 0.13	2.81	0.73	10.79

**Table 6:** Summary of comparative perfusion parameters for the benign and malignant groups.

		Lesion/Parenchyma Ratio			
DCE-US Parameters	Diagnosis	Mean	Std. Deviation	Std. Error Mean	*p*-Value (Benign vs. Malignant)
TTP	Benign	0.85	0.18	0.05	*p =* 0.41
	Malignant	0.89	0.21	0.04	
PV	Benign	11.77	18.73	5.70	*p =* 0.73
	Malignant	10.08	22.09	3.68	
WIT	Benign	2.13	2.69	0.72	*p =* 0.25
	Malignant	2.06	4.88	0.81	
WOT	Benign	1.02	0.72	0.19	*p =* 0.98
	Malignant	1.64	4.27	0.71	
Inflow Rate	Benign	13.38	27.92	7.46	*p =* 0.49
	Malignant	22.44	73.66	12.28	
Inflow Rate (5 s)	Benign	10.86	18.41	4.92	*p =* 0.56
	Malignant	12.95	20.00	3.50	
Outflow Rate	Benign	18.84	37.84	10.11	*p =* 0.13
	Malignant	18.50	40.83	7.31	
Outflow Rate (5 s)	Benign	17.51	21.91	5.86	*p =* 0.12
	Malignant	18.87	23.37	8.57	
MTT	Benign	1.07	0.80	0.21	*p =* 0.52
	Malignant	1.10	0.68	0.11	

## Data Availability

The datasets presented in this article are unavailable due to privacy restrictions.
